# Genetic mapping identifies genomic regions and candidate genes for seed weight and shelling percentage in groundnut

**DOI:** 10.3389/fgene.2023.1128182

**Published:** 2023-03-16

**Authors:** Sunil S. Gangurde, Janila Pasupuleti, Sejal Parmar, Murali T. Variath, Deekshitha Bomireddy, Surendra S. Manohar, Rajeev K. Varshney, Prashant Singam, Baozhu Guo, Manish K. Pandey

**Affiliations:** ^1^ Center of Excellence in Genomics & Systems Biology (CEGSB), International Crops Research Institute for the Semi-Arid Tropics (ICRISAT), Hyderabad, India; ^2^ Department of Genetics, Osmania University, Hyderabad, India; ^3^ USDA-ARS, Crops Genetics and Breeding Research Unit, Tifton, GA, United States; ^4^ State Agricultural Biotechnology Centre, Centre for Crop & Food Innovation, Food Futures Institute, Murdoch University, Murdoch, WA, Australia

**Keywords:** candidate gene discovery, peanut, diagnostic markers, high-density genotyping, Axiom_*Arachis* array

## Abstract

Seed size is not only a yield-related trait but also an important measure to determine the commercial value of groundnut in the international market. For instance, small size is preferred in oil production, whereas large-sized seeds are preferred in confectioneries. In order to identify the genomic regions associated with 100-seed weight (HSW) and shelling percentage (SHP), the recombinant inbred line (RIL) population (Chico × ICGV 02251) of 352 individuals was phenotyped for three seasons and genotyped with an Axiom_*Arachis* array containing 58K SNPs. A genetic map with 4199 SNP loci was constructed, spanning a map distance of 2708.36 cM. QTL analysis identified six QTLs for SHP, with three consistent QTLs on chromosomes A05, A08, and B10. Similarly, for HSW, seven QTLs located on chromosomes A01, A02, A04, A10, B05, B06, and B09 were identified. *BIG SEED* locus and *spermidine synthase* candidate genes associated with seed weight were identified in the QTL region on chromosome B09. Laccase, fibre protein, lipid transfer protein, senescence-associated protein, and disease-resistant NBS-LRR proteins were identified in the QTL regions associated with shelling percentage. The associated markers for major-effect QTLs for both traits successfully distinguished between the small- and large-seeded RILs. QTLs identified for HSW and SHP can be used for developing potential selectable markers to improve the cultivars with desired seed size and shelling percentage to meet the demands of confectionery industries.

## 1 Introduction

Groundnut or peanut is a self-pollinated, allotetraploid (AABB) (2n = 4X = 40), and leguminous oilseed crop with ∼2.7 GB genome size (Bertioli et al., 2019). Presently, groundnut is cultivated globally on 36.18 million hectares of area, yielding 71.68 million tonnes of pods in the year 2020 (FAOSTAT, 2020). Groundnut seeds contain the most nutritious components; 100 g of groundnuts contains proteins (16 g), oil or fat (49 g), carbohydrate (26 g), and dietary fibres (9 g) (Parmar et al., 2022). The estimated demand of edible oil based on current population projections and *per capita* consumption is likely to be 240 metric tonnes by 2050, which is nearly twice the current requirement (Corley, 2009). Improvement of yield and quality traits are the major objectives of many groundnut breeding programs (Gangurde et al., 2019; Pandey et al., 2020a). Groundnut yield is influenced by hundred-seed weight (HSW), shelling percentage (SHP), and number of seeds per pod. Among them, HSW and seed number per pod are important allied traits that are positively correlated with groundnut yield. HSW is an important yield attributing trait that is positively correlated with yield per plant. Being a quantitative trait, seed weight is controlled by multiple genes and also influenced by the environment. Therefore, understanding the regulation of seed size has always been an important area of research for groundnut improvement.

In the pre-genomic era, efforts on genetic mapping for seed weight produced large QTL intervals, making identification of the key candidate genes very difficult (Varshney et al., 2009; Mondal & Badigannavar, 2019). QTLs for seed size and pod size in cultivated and wild relatives of groundnut were discovered using an advanced backcross population (Fonceka et al., 2012). Consistent QTLs for seed weight on chromosome A07 and B06 and for shelling percentage on A10 and B06 were identified using an SSR-based genetic map (Chen et al., 2017). During the last few years, since the availability of groundnut diploid genomes (Bertioli et al., 2016; Chen et al., 2016) and tetraploid genomes (Bertioli et al., 2019; Chen et al., 2019; Zhuang et al., 2019), several sequencing-based trait mapping efforts have been reported to fine map the genomic regions for key traits in groundnut. The discovered candidate genes include those associated with leaf rust and late leaf spot resistance (Pandey et al., 2017a), stem rot resistance (Dodia et al., 2019), fresh seed dormancy (Kumar et al., 2020), shelling percentage (Luo et al., 2019a), bacterial wilt resistance (Luo et al., 2019b), early leaf spot and late leaf spot resistance (Agarwal et al., 2018), tomato spotted wilt virus resistance (Agarwal et al., 2019), and yield-related traits (Jadhav et al., 2021). In the post-genomic era, a significant amount of genomic resources at the genome and transcriptome level have been developed in groundnut (Pandey et al., 2020a). The transcriptome map for subsp. *hypogaea* was developed to understand the differential expression of genes at various growth stages (Clevenger et al., 2016). Moreover, another gene expression atlas for subsp. *fastigiata* was developed for 20 tissues at various developmental stages, ranging from the seedling stage to the maturity stage (Sinha et al., 2020).

The development of high-density SNP chips in groundnut allowed the construction of high-density genetic maps that helped to saturate the large QTL intervals (Pandey et al., 2017b). SNP arrays were successfully used to dissect the yield-related traits (Pandey et al., 2020b), root-knot nematode resistance (Ballén-Taborda et al., 2019), stem rot resistance (Luo et al., 2020), late leaf spot resistance (Han et al., 2018; Chu et al., 2019; Zhang et al., 2020), fresh seed dormancy (Wang et al., 2022), leaf chlorophyll content (Zou et al., 2022), salinity tolerance (Zou et al., 2020a), background genome recovery during a marker-assisted backcross selection in groundnut (Shasidhar et al., 2020), and germplasm diversity analysis (Nabi et al., 2021). Specific locus amplified fragment sequencing (SLAF-seq)-based high-density genetic map and phenotyping under multiple environments identified a total of 27 QTLs for seed weight, seed length, and width (Zhang et al., 2019). Recently, US-based nested association mapping (NAM) populations genotyped with a 58K SNP array discovered the genomic regions associated with seed and pod weights in groundnut (Gangurde et al., 2020). The SSR and SNP array-based genetic map identified the major genomic region on chromosome B06 and homologous region on chromosome A07/B07 (Chavarro et al., 2020). It has also been reported that in the US mini-core collection, the QTL on chromosome A05 is conserved with major effects on groundnut seed size (Chu et al., 2020). Similarly, in Chinese germplasm, the QTL on chromosome A05 was identified for the seed number per pod (Chen et al., 2019). Recently, the 58K SNP array was used to identify genomic regions associated with the seed aspect ratio (length width ratio) using GWAS on the US mini-core and Korean germplasm (Zou et al., 2020b). Although a large number of studies have been conducted globally to identify the QTLs for groundnut seed weight, there are limited reports on candidate genes or diagnostic markers for genomic-assisted breeding to improve seed weight.

Therefore, in order to identify the genomic regions and candidate genes associated with HSW and SHP, we developed a recombinant inbred line (RIL) population (Chico × ICGV 02251). The parental genotypes included a large-seeded cultivar “ICGV 02251” and a small-seeded germplasm line “Chico.” A 58K high-density SNP array was used to genotype the RIL population along with the two parents to construct a dense genetic map. The genetic map along with genotyping data and multiple seasons phenotyping data was used to identify the genomic regions associated with HSW and SHP in groundnut.

## 2 Materials and methods

### 2.1 Plant material and phenotyping

A RIL population (Chico × ICGV 02251) comprising 352 RILs was developed by crossing Chico and ICGV 02251 and advanced using the single-seed decent (SSD) method. Both parents were allotetraploid (AABB), *Arachis hypogaea* and subsp. *fastigiata*, and the male parent, ICGV 02251, a late-maturing, Virginia bunch, has a significantly higher HSW and larger pod size than the female parent. Chico is an early-maturing Spanish bunch; a selection from PI 268661 was released in 1973 by the United States Department of Agriculture (USDA) in Georgia, Virginia, and Oklahoma (Bailey and Hammons, 1975). The RIL population was phenotyped for three seasons at ICRISAT, Patancheru, Hyderabad (India) during post-rainy 2013–14 (S1), rainy 2014 (S2), and rainy 2019 (S3). During each season, the RILs and parental lines were planted in three replications with a spacing of 30 × 10 cm in two rows of 2 m, with standard agronomic practices. The weather data for monthly average high and low temperatures and rainfall (mm) for the years 2013, 2014, and 2019 are shown in Supplementary Figure S1. The weight of 100 mature groundnut seeds from each RIL was measured as hundred-seed weight (gm), while for shelling percentage, 100 gm pods were shelled and the seed weight from these pods (gm) was divided by pod’s weight and multiplied by 100 as a measure of the shelling percentage (%). The multi-season phenotypic data for SHP and HSW on 352 RILs and both parents were used for the identification of genomic regions associated with HSW and SHP.

### 2.2 DNA extraction and genotyping with an “Axiom_*Arachis*” array

DNA from 352 RILs and both parents was extracted using the NucleoSpin Plant II kit (Macharey-Nigel, Duren, Germany). The DNA quality was analysed on 0.8% agarose gel, and concentration was measured using a NanoDrop 8000 spectrophotometer (Thermo Scientific). An Affymetrix GeneTitan® platform was used to genotype the RIL population with the 58K SNPs “Axiom_*Arachis*” array (Pandey et al., 2017b). Initially, the target probes for 352 samples were used in at least 20 µL DNA, with a concentration of 10 ng/µL. The samples were then amplified, fragmented, and hybridized on the array chip, followed by single-base extension through DNA ligation and signal amplification, according to the procedure explained in the Affymetrix Axiom 2.0 Assay manual (axiom_2_assay_auto_workflow_user_guide.pdf) (Pandey et al., 2020a). Axiome_*Arachis* is an SNP array developed for genotyping genetic populations in groundnut for trait mapping and association mapping (Pandey et al., 2017b).

### 2.3 SNP allele calling and quality analysis

We used the “*Best Practices*” workflow to perform quality control (QC) analysis of samples to select only those that pass the QC test for further downstream analysis. The “*Sample QC*” workflow was used to produce genotype calls for the samples that passed the QC test. The “*Genotyping*” workflow was used to perform genotyping on the imported CEL files regardless of the sample QC matrix. Before making the genotyping calls, samples that did not pass the QC were removed as their inclusion may reduce the quality of the analysed results. Finally, the “*Summary Only*” workflow was used to produce a summary containing details on the intensities for the probe sets for use in copy number analysis tools. It also allows exporting the SNP data after the analysis is completed for downstream analysis. The genotyping data with a towere filtered for monomorphismtal of 58,233 SNPs for 352 RILs was extracted from Axiom analysis suit as explained in Pandey et al., 2017b (Supplementary Table S1).

### 2.4 Construction of a genetic map using RIL population (Chico × ICGV 02251)

The 58,233 SNPs were filtered for monomorphism and highly missing (>30%), and only the selected polymorphic 10,236 SNPs between parental genotypes, ICGV 02251, and Chico were retained. The selected SNPs were subjected to the chi-square (χ^2^) test to determine the goodness-of-fit to the expected 1:1 segregation ratio; highly distorted markers were filtered out and not considered for the linkage map construction. Finally, after stringent filtration, informative SNPs were used for the construction of a genetic map. The alleles of ICGV 02251 were coded as “AA,” Chico as “BB,” and heterozygotes as “H.” JoinMap (v4.0) software was used for the construction of a genetic map. Kosambi’s mapping function was used to estimate the genetic distance to convert the recombination frequencies into map distances in centimorgans (cMs) (Kosambi, 1944). A total of 20 linkage groups were constructed individually by applying the LOD score (logarithm of the odds) with an LOD threshold of 3.0, and the recombination frequency (*rf*) threshold (∂) was set to 50%. MapChart software was used to finalize the marker position with the final genetic map (Voorrips, 2002).

### 2.5 Identification of QTLs for seed weight and shelling percentage

Multi-season phenotyping data for seed weight and shelling percentage generated during S1, S2, and S3 was used with genetic map information along with genotyping data for QTL analysis. The inclusive composite interval mapping-additive (ICIM-ADD) algorithm implemented in inclusive composite interval mapping (ICIM) software was used for identification of the main-effect QTLs (Meng et al., 2015). Epistatic QTLs for seed weight and shelling percentage were identified to understand the combined effect of any two genomic regions on seed weight and shelling percentage. QTLs with >10% phenotypic variance explained (PVE) were considered major QTLs; the remaining were considered minor QTLs. Realizing that the groundnut seed weight is a complex trait and also affected by the environment, we carried out epistatic QTL (Q × Q) and environment effect QTL (E-QTL) analysis in ICIM. The ICIM-EPI algorithm from ICIM software was used for epistatic QTL analysis. The environmental-effect QTLs were identified using multi-environment trails (METs) in ICIM. The LOD threshold score of 3.0 was used as the minimum significance level for the main effect QTLs, epistatic QTLs, and environmental-effect QTLs. The major QTL regions were validated using extreme RILs for both HSW and SHP from RIL population. A total of 15 extremely small-seeded and 15 extremely large-seeded lines were used for the validation of QTL regions. In order to validate the major QTLs, the RILs distinguished by the alleles of the flanking markers of QTLs were compared with the mean values of phenotypes.

### 2.6 Identification and expression analysis of candidate genes in QTL regions

Major QTL regions identified with >10% PVE identified for both seed weight and shelling percentage were targeted for candidate gene discovery. Candidate genes associated were mined in the QTL interval between the positions of flanking markers on the physical map of groundnut genome (https://peanutbase.org/). The expression data for the candidate genes were accessed from the *Arachis hypogaea* gene expression atlas for subsp. *fastigiata* (Sinha et al., 2020). The heatmaps for the expression data were generated using the R-software package “Pheatmap” (Kolde, 2019).

## 3 Results

### 3.1 Phenotypic variation for seed weight and shelling percentage in RIL population

**FIGURE 1:**
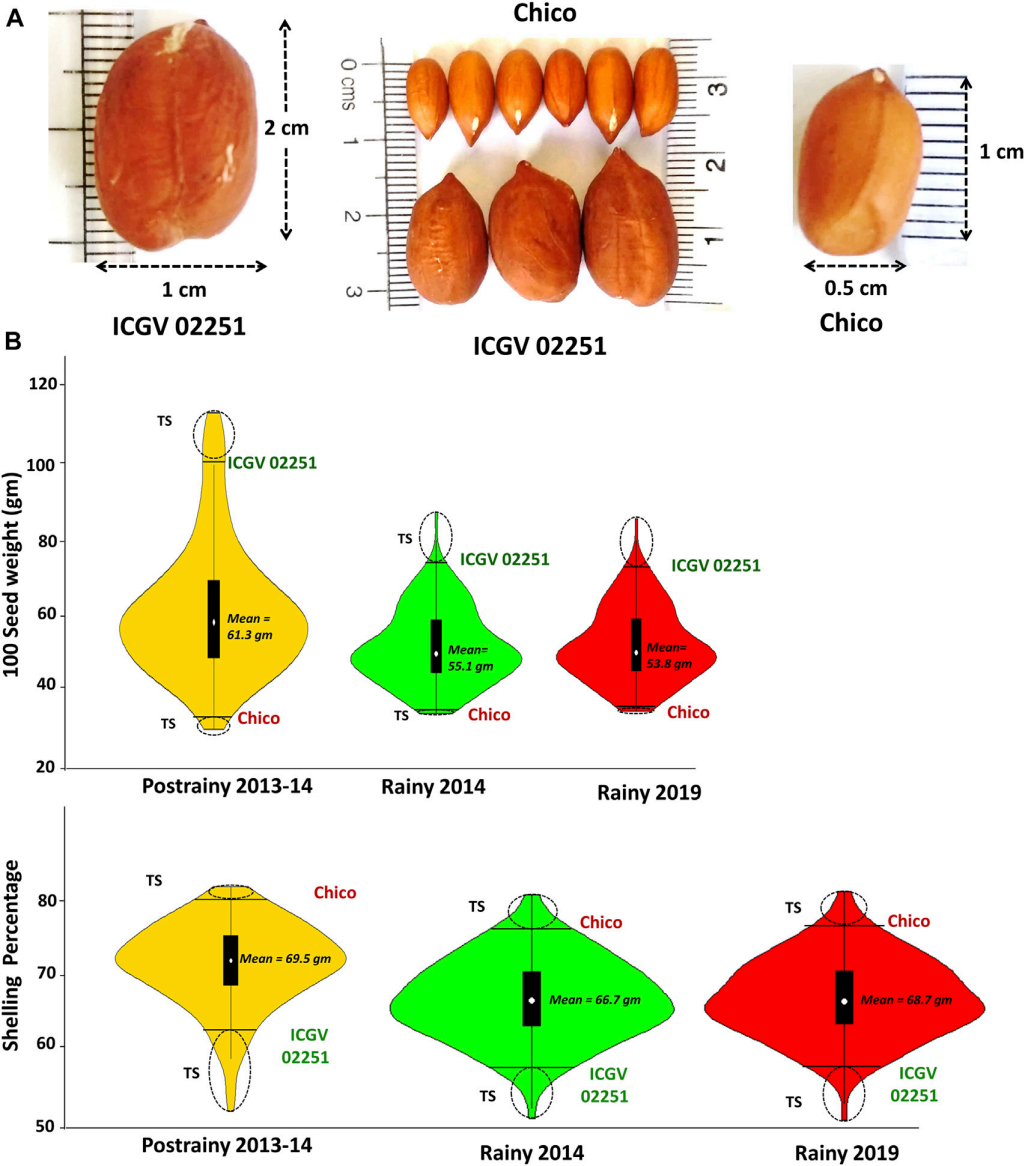
Variation in the phenotypic data generated for **(A)** hundred seed weight and **(B)** shelling percentage in the RIL population (Chico × ICGV 02251) during three seasons (Postrainy 2013-14, Rainy 2014 and Rainy 2019).

Multi-season phenotyping data was generated during three seasons on RIL population (Chico × ICGV 02251). ICGV 02251 was used as the male parent with HSW of 101.8 gm, while Chico (small-seeded cultivar) was used as a female parent with HSW of 35.0 gm. Furthermore, Chico has a high SHP with 80.5%, whereas ICGV 02251 (large-seeded cultivar) had a medium SHP of 69.5%. The average HSW in RIL population was 61.5 gm during S1, 43.5 gm during S2, and 45.5 gm during S3, whereas the average SHP was 69.40% during S1, 66.7% during S2, and 68.75% during S3. The seed weight and shelling percentage were higher in season S1 than those in S2 and S3. The phenotypic data generated in three seasons for both HSW and SHP showed normal distribution on violin plots (Figures 1A, B).

### 3.2 Important features of SNP array-based genetic map

**FIGURE 2:**
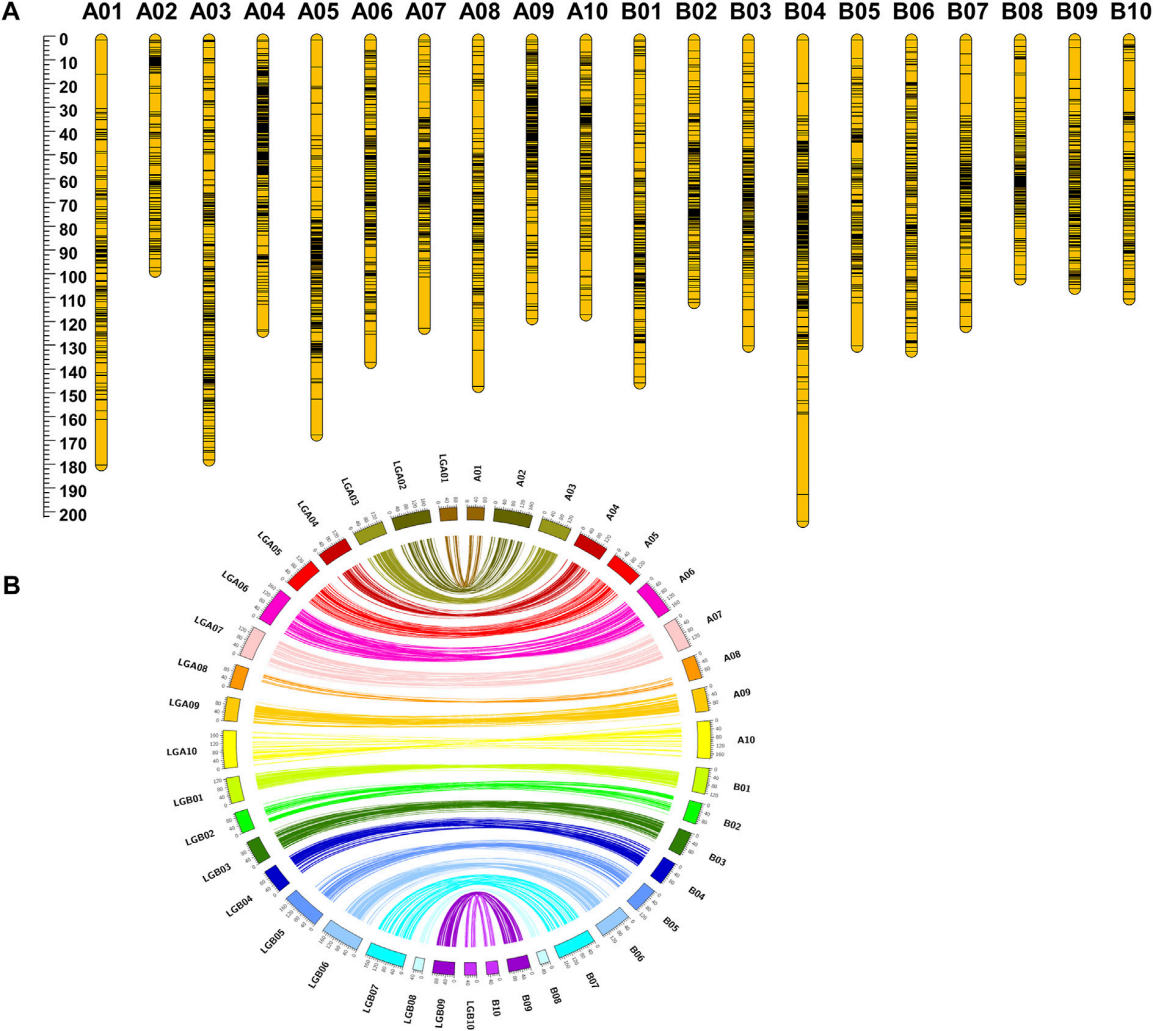
Highly collinear genetic map constructed using RIL population Chico × ICGV 02251. **(A)** SNP loci density on the 20 linkage groups. **(B)** Collinearity of the genetic map with the physical map.

The filtered 6235 SNPs were used for genetic map construction. A total of 4199 loci were mapped on A and B subgenomes with a total distance of 2708.36 cM. A total of 2036 SNPs did not show any linkage with the SNP markers in the generated genetic map. No attempt was made to map the unlinked SNPs in the final genetic map to avoid noise during QTL analysis. Among the 4199 SNP loci, 2343 loci were mapped on A subgenome, whereas 1856 loci were mapped for B subgenome with a distance of 1456.96 cM and 1251.4 cM, respectively. The A and B subgenomes reached an average inter-marker distance of 0.66 and 0.67 cM/loci, respectively. A maximum number of loci mapped in a specific linkage group ranged from 128 (B10) to 447 (A04). The average inter-marker distance for each linkage group ranged from 97.34 cM (A02) to 202.5 cM (B04). The average inter-marker distance was maximum, 1.17 cM/loci, for the linkage group A01 and minimum, 0.47 (cM/loci), for the linkage group A09 (Table 1; Figure 2).

**TABLE 1:** Summary of a genetic map constructed based on RIL population Chico × ICGV 02251.

Linkage group	Total loci	Mapped loci	Length of LG (cM)	Average inter-marker distance (cM/loci)	Linkage group	Total loci	Mapped loci	Length of LG (cM)	Average inter-marker distance (cM/loci)
A subgenome					B subgenome				
A01	4714	153	178.79	1.17	B01	2405	176	114.3	0.65
A02	3167	159	97.34	0.61	B02	3112	184	110.5	0.60
A03	3478	267	176.65	0.66	B03	3443	212	128.8	0.61
A04	2693	447	202.5	0.64	B04	2588	315	202.5	0.64
A05	2624	228	166.17	0.73	B05	2576	162	128.8	0.80
A06	2764	270	135.57	0.50	B06	2793	198	131	0.66
A07	2303	234	121.34	0.52	B07	2638	162	120.5	0.74
A08	2921	168	145.5	0.87	B08	2671	145	100.6	0.69
A09	2790	249	117.3	0.47	B09	3152	174	104.9	0.60
A10	2529	168	115.8	0.69	B10	2872	128	109.5	0.86
Total	29983	2343	1456.96	0.66	Total	28250	1856	1251.4	0.67
Grand total	58233	4199	2708.36	0.65					

### 3.3 Identification of the major-effect QTLs associated with seed weight and shelling percentage

A total of 13 major QTLs were identified for HSW and SHP; six QTLs were identified for SHP with 5.3%–15.8% PVE and LOD score ranging from 2.51 to 7.16 during three seasons (S1, S2, and S3); and seven QTLs were identified for HSW with 6.96%–21.29% PVE and LOD score ranging from 3.9 to 11.7 during three seasons (Table 2).

**TABLE 2:** Main effect of QTLs for hundred-seed weight and shelling percentage.

QTL name	Season	Chr	Pos	Left marker	Right marker	LOD	PVE (%)	ADD	Contributing parent
**Shelling percentage (SHP)**									
*qShP-A04.1*	S1	A04	122	A04_59857421	A04_22131549	2.51	5.32	−2.10	Chico
*qShP-A05.1*	S2	A05	82	A05_22902580	A05_97942457	3.54	11.00	−2.42	Chico
	S3	A05	82	A05_22902580	A05_97942457	3.62	11.07	−2.41	Chico
*qShP-A08.1*	S3	A08	83	A08_43239201	A08_34847702	3.81	9.41	−2.04	Chico
	S2	A08	83	A08_43239201	A08_34847702	3.81	9.41	−2.04	Chico
*qShP-B04.1*	S1	B04	156	B04_130804224	B04_112728451	3.49	9.11	−1.98	Chico
*qShP-B06.1*	S1	B06	46	B06_125401834	B06_122810153	7.16	15.80	−2.68	Chico
*qShP-B10.1*	S3	B10	78	B10_135814931	B10_132665342	4.73	12.97	2.40	ICGV 02251
	S2	B10	78	B10_135814931	B10_132665342	4.64	12.80	2.39	ICGV 02251
**Hundred-seed weight (HSW)**									
*qHSW-A01.1*	S1	A01	152	A01_95668523	A01_104923102	8.82	20.65	10.25	ICGV 02251
*qHSW-A02.1*	S1	A02	40	A02_1992802	A02_84637409	4.20	6.96	4.79	ICGV 02251
*qHSW-A04.1*	S1	A04	61	A04_118815935	A04_33608076	8.01	14.49	3.53	ICGV 02251
	S3	A04	61	A04_118815935	A04_33608076	11.67	17.12	4.19	ICGV 02251
*qHSW-A10.1*	S2	A10	89	A10_109094772	A10_104674897	3.98	10.68	2.22	ICGV 02251
*qHSW-B05.1*	S2	B05	39	B05_25495898	B05_26588237	7.66	21.29	3.39	ICGV 02251
*qHSW-B06.1*	S3	B06	54	B06_112472327	B06_110468072	7.13	13.65	3.17	ICGV 02251
*qHSW-B09.1*	S1	B09	44	B09_145481859	B09_146042834	6.85	12.00	5.78	ICGV 02251

**S1**, Post-rainy 2013–14; **S2**, rainy 2014; **S3**, rainy 2019; **Chr**, chromosome; **Pos**, position; **LOD**, logarithm of odds; **PVE**, phenotypic variance explained; **ADD**, additive effect.

### 3.4 QTLs identified for shelling percentage

**FIGURE 3:**
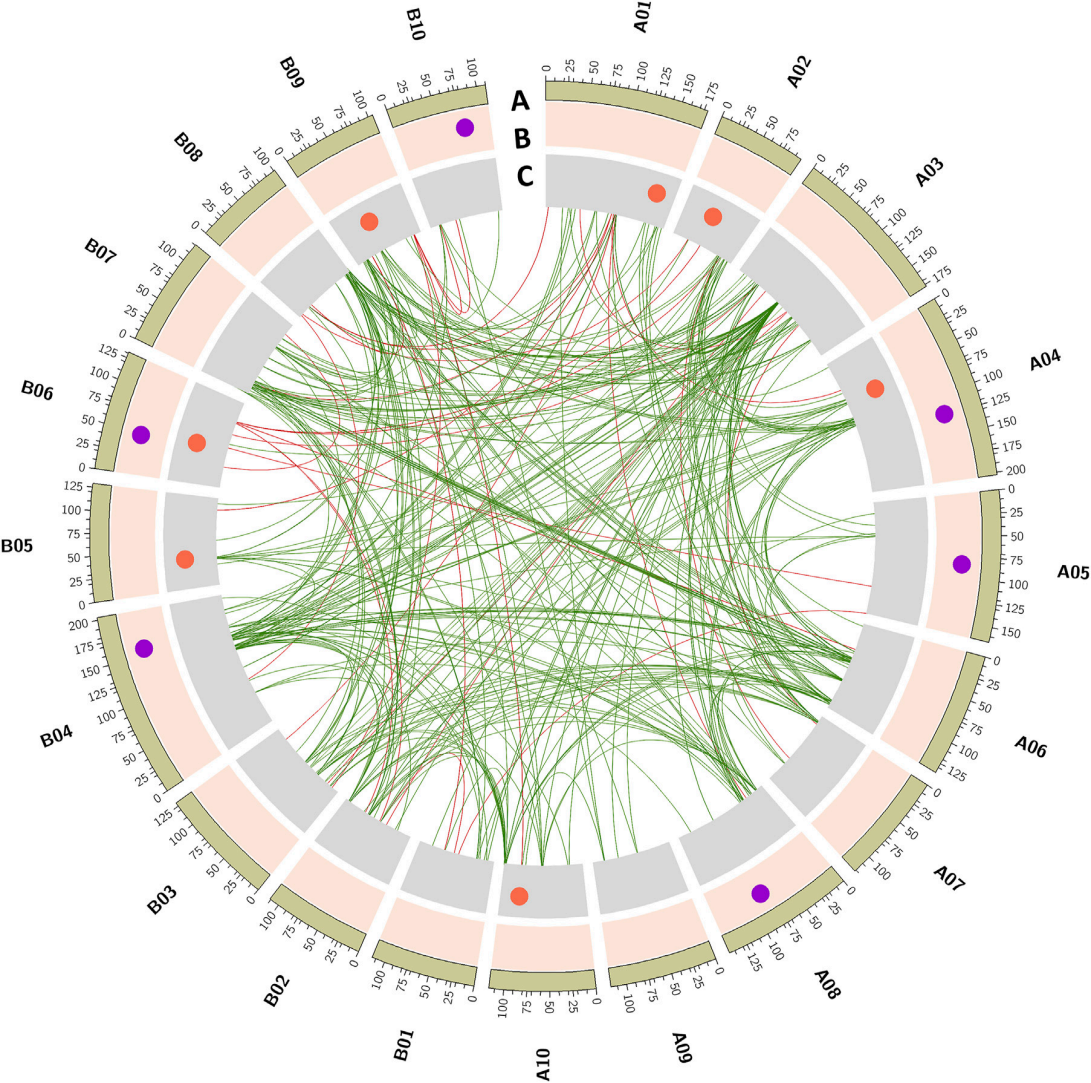
Main-effect QTLs identified for hundred-seed weight (HSW) and shelling percentage (SHP). The tracks outside to inside illustrates, **(A)** 20 chromosomes of cultivated groundnut labeled as A01 to A10 and B01 to B10, **(B)** QTLs identified for shelling percentage (SHP), **(C)** QTLs identified for hundred seed weight. Inner links represent epistatic (Q × Q) interactions. Green color links represent the epistatic interaction for shelling percentage, and red color links are epistatic interactions for seed weight.

Of the six QTLs identified for SHP, a QTL identified on chromosome A04 (*qShP-A04.1*) showed a minor effect of 5.32% PVE. A QTL was consistently detected on A05 (*qShP-A05.1*) with LOD score of 3.5 and 11.07% PVE in seasons S2 and S3, respectively. A QTL *qShP-A08.1* on A08 with LOD score of 3.81 and 9.41% PVE was also consistently identified in seasons S2 and S3. A QTL (*qShP-B04.1*) identified on B04 with 9.11% PVE and *qShP-B06.1* identified on B06 with 7.16 LOD and 15.80% PVE. A QTL *qShP-B10.1* identified on B10 with LOD score of 4.64 and 12.8% PVE was consistently identified in seasons S2 and S3 (Table 2; Figure 3).

### 3.5 QTLs for hundred-seed weight

A total of seven QTLs were identified for HSW with a PVE range of 6.9%–21.29% and LOD score ranging from 3.9 to 11.7. A single QTL (*qHSW-A01.1*) was identified on A01 with 20.65% PVE and 8.82 LOD. On A02, a minor QTL (*qHSW-A02.1*) was identified with 4.2 LOD and 6.96% PVE. A major effect QTL *qHSW-A04.1* was identified on A04 with ∼17.12% PVE in both seasons S1 and S3. Two QTLs were identified in S2, *qHSW-A10.1* on A10 with 3.98 LOD and 10.68% PVE and *qHSW-B05.1* on B05 with 7.66 LOD and 21.29% PVE. A major-effect QTL (*qHSW-B09.1*) was detected on B09 with 6.85 LOD and 12.0% PVE. A QTL *qHSW-B06.1* on B06 explained 13.65% PVE (Table 2; Figure 3).

### 3.6 Identification of epistatic interactions for seed weight and shelling percentage

A total of 375 E-QTLs were identified for shelling percentage and seed weight-related traits. A total of 42 E-QTLs were detected for SHP and 332 E-QTLs for HSW. The phenotypic variation explained by the E-QTLs identified for seed weight and shelling percentage ranged from 10.0%–11.2% to 10.58%–27.09%, respectively.

A total of 332 epistatic QTLs were detected for HSW, of which 14 epistatic QTLs were identified with 3.60–7.32 LOD score and 10.02%–16.28% PVE during S2. On the other hand, 37 epistatic QTLs were identified with 3.60–6.91 LOD score and 10.02%–16.28% PVE during S1. A total of 182 epistatic QTLs were identified with 3.61–6.28 LOD, 10.20%–11.12% PVE during S3, of which 29 major epistatic QTLs were identified (LOD > 5.0) for HSW.

A total of 42 epistatic QTLs were detected for shelling percentage, and among them, eight had 3.05–3.55 LOD score and 10.65%–27.09% PVE during S1. The rest of the QTLs included 18 epistatic QTLs with 3.03–5.47 LOD score and 10.58%–26.31% PVE during S2 and 18 epistatic QTLs with 3.01–5.26 LOD score and 10.86%–25.14% PVE during S3. Major epistatic QTL for shelling percentage was identified with PVE of 15.46% and an LOD score of 5.4 that showed the interaction between genomic regions of chromosomes A02 and B06 (Figure 3; Supplementary Table S2).

### 3.7 Identification of environmental effect QTLs (Q × E) for seed weight and shelling percentage

**FIGURE 4:**
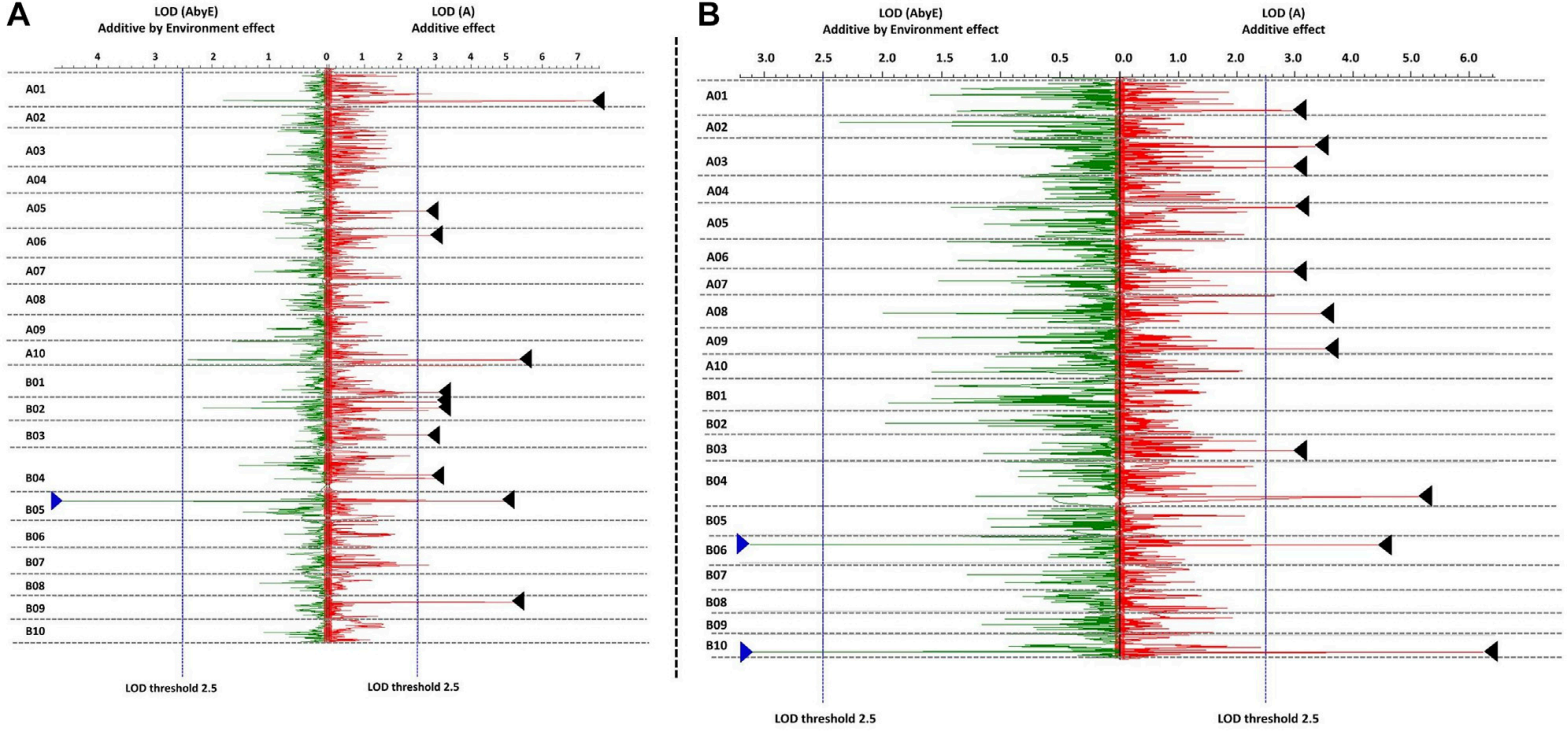
Main effect QTLs with major environment effect identified for **(A)** hundred seed weight, and **(B)** shelling percentage (%). Blue triangles represent additive by environment effect and black triangles represent the effect due genetic background.

A total of 15 environmental-effect QTLs were identified for seed weight and shelling percentage with LOD score value > 3.0. A total of seven environmental-effect QTLs were identified for seed weight with 4.11–9.56 LOD scores and 4.81%–27.18% PVE. A major environmental-effect QTL (*EqtlSW.B05.1*) on B05 was identified for seed weight with PVE of 10.03%. The same QTL region (*qHSW-B05.1*) was also identified as a main-effect QTL for HSW (Figure 4A). A total of three such E-QTLs for HSW showed high PVE on chromosome A01 (27.1%), B05 (10.0%), and B09 (12.3%). Here, we concluded that the QTL region on chromosome B05 explains the higher phenotypic variance due to the environmental effect with partial effects from a background genome. A total of eight environmental-effect QTLs were identified for SHP with 3.68–9.32 LOD score and 3.96%–11.06% PVE. A total of two major E-QTLs for SHP each on chromosome B06 and B10 were identified with PVE of 11.06% on B10 (Figure 4B). We plotted QTL additive effects against additive by environmental effects to find the QTLs which are highly influenced by the environment. We observed that both SHP and HSW showed higher (>10%) phenotypic variance explained by environmental effects (Supplementary Table S3).

### 3.8 Candidate genes for hundred-seed weight and shelling percentage

**FIGURE 5:**
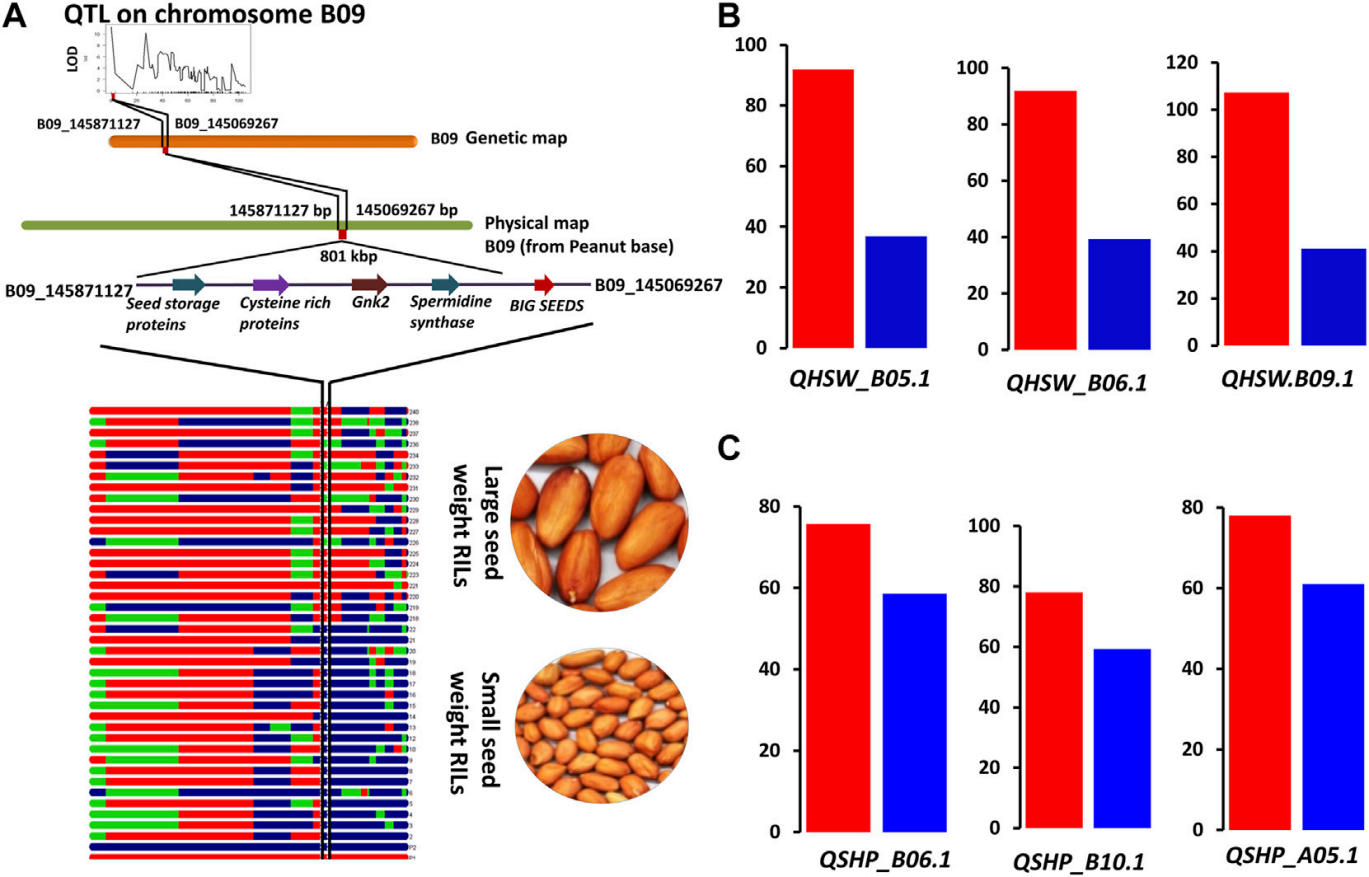
Validation of QTL regions using RILs for seed weight and shelling percentage. **(A)** QTL region and candidate genes on chromosome B09 associated with groundnut seed weight, **(B)** validation of main-effect QTLs for hundred-seed weight (HSW). **(C)** Validation of QTLs for shelling percentage (SHP). Red bars represent average shelling percentage of high-shelling percentage RILs, and blue bars represent average shelling percentage of low shelling percentage RILs.

QTLs for both HSW and SHP with PVE >10.0 were targeted for candidate gene discovery. With this criterion, a total of three QTLs for HSW, namely, *qHSW_B05.1*, *qHSW_B06.1*, and *qHSW_B09.1* were investigated for candidate gene discovery. In the genomic region of *qHSW_B05.1* (0.9 Mb), a total of 16 candidate genes including nodulin MtN21 (*Araip.IJC5B*), acyl-CoA acyltransferase (*Araip.FGM9R*), yellow stripe-like (YSL) proteins (*Araip.Y0XXQ*), and serine–threonine protein phosphatase (*Araip.QEE5K*) were identified. A total of 47 genes were identified in the QTL region *qHSW_B06.1* (2.0 Mb) on chromosome B06. The key genes in this region included zinc-finger proteins (*Araip.DYC48*), MYB transcription factors (*Araip.6N0ZN*), receptor-like serine/threonine kinases (*Araip.QR111*), E3 ubiquitin–protein ligase (*Araip.R3W9S*), methyltransferase-like protein (*Araip.YM7ML*), and glutamate dehydrogenase (*Araip.Q3F5T*). A total of 51 candidate genes were identified in the QTL region *qHSW_B09.1* (0.5 Mb) on chromosome B09 (Figure 5A). In this region, an important candidate gene from TIFY family proteins called *BIG SEED* locus was identified (*Araip.YK09Y*) along with *spermidine synthase* (*Araip.8Z3VM*), seed linoleate lipoxygenase (*Araip.D6PZJ*), epidermal patterning factor (*Araip.9SE5V*), cytochrome P450 superfamily protein (*Araip.A4X5Z*), and sugar transporters (*Araip.4HV2H*) (Figure 5B; Supplementary Table S4).

Similarly, for shelling percentage, we targeted three main major effect QTLs, namely, *qShP_A05.1*, *qShP_A08.1*, and *qShP_B10.1* (Figure 5C). The QTL on chromosome A05 (*qShP_A05.1*) harboured 47 candidate genes including *spermidine synthase* (*Aradu.FH71M*), receptor-like protein kinases (*Aradu.CU9HL*), disease-resistance protein (*Aradu.G0IJA*), Fe superoxide dismutase (*Aradu.3HY3W*), Ubiquitin–protein ligase, FAR1-related sequence 5-like (*Aradu.EW4Y3*), and tetratricopeptide repeat protein (*Aradu.1L0UP*). A total of 120 genes were identified in the QTL region (*qShP_A08.1*) on chromosome A08. The genes in this region included C_2_H_2_ zinc-finger protein (*Aradu.8HA7W*), cellulose synthase family protein (*Aradu.8HA7W*), chilling-induced protein (*Aradu.F7DPM*), defensin (*Aradu.CK6H7*), disease-resistance protein (TIR-NBS-LRR) (*Aradu.HLR71*), SUMO proteins (*Aradu.3IM1W*), fibre protein (*Aradu.3IM1W*), MYB transcription factor, NAC domain, senescence-associated proteins (*Aradu.GR4IB*), and Ulp proteases (*Aradu.G32CD*). A total of 84 genes were identified in the QTL region (*qShP_B08.1*) on chromosome B08. The candidate genes in this region included calcium-dependent lipid-binding protein (*Araip.B3EI3*), cellulose synthase (*Araip.U1RD3*), sugar transporters (SWEET) (*Araip.3H2LW*), Rubisco methyltransferase family protein (*Araip.N03N5*), disease-resistance protein (*Araip.P42EJ*), and glycine-rich abscisic acid inducible gene (*Araip.MZ5SZ*) (Table 3; Supplementary Table S4).

**TABLE 3:** Potential candidate genes identified for hundred-seed weight and shelling percentage in major QTL regions.

**QTL**	**Chromosome**	**Gene ID**	**Start**	**End**	**Annotation**
***QShP_A05.1***	Aradu.A05	*Aradu.CU9HL*	22986869	22990293	Receptor-like protein kinase
	Aradu.A05	*Aradu.B4D3A*	24879878	24881965	U-box domain-containing protein
	Aradu.A05	*Aradu.MYH5B*	24792286	24793231	Adaptor protein complex
	Aradu.A05	*Aradu.FH71M*	24920996	24925015	Spermidine synthase
	Aradu.A05	*Aradu.G0IJA*	24901015	24904320	Disease resistance protein
	Aradu.A05	*Aradu.3HY3W*	24892006	24900621	Fe superoxide dismutase
	Aradu.A05	*Aradu.H04NN*	23876875	23880528	Protein kinase superfamily protein
	Aradu.A05	*Aradu.48LC5*	23381620	23385794	ATP-citrate lyase
	Aradu.A05	*Aradu.D08CT*	23649272	23653365	E3 ubiquitin-protein ligase
	Aradu.A05	*Aradu.Y354W*	23590437	23591175	Serine/threonine-protein phosphatase
	Aradu.A05	*Aradu.AP3A5*	23740398	23740895	GRF zinc finger protein
***QShP_A08.1***	Aradu.A08	*Aradu.6G4CM*	37286958	37292277	Calcium-binding EF-hand family protein
	Aradu.A08	*Aradu.440M4*	37377596	37378358	Defensin related
	Aradu.A08	*Aradu.HLR71*	43189988	43192287	Disease resistance protein (TIR-NBS-LRR class)
	Aradu.A08	*Aradu.7I425*	36999018	37001500	Fiber protein
	Aradu.A08	*Aradu.QL5QA*	41446652	41447645	Laccase
	Aradu.A08	*Aradu.V4L4B*	37275421	37281934	Leucine-rich repeat receptor-like protein
	Aradu.A08	*Aradu.FXW2H*	38389997	38390929	Lipid transfer protein
	Aradu.A08	*Aradu.AK182*	37321258	37328555	MLO-like protein 11-like
	Aradu.A08	*Aradu.888CN*	38843334	38844343	MYB transcription factor MYB82
	Aradu.A08	*Aradu.USH95*	38011876	38013744	NAC domain protein
	Aradu.A08	*Aradu.GR4IB*	42739667	42742321	Senescence-associated protein
	Aradu.A08	*Aradu.G32CD*	42492569	42494401	Ulp1 protease family C-terminal catalytic domain containing protein n
	Aradu.A08	*Aradu.2X4SQ*	39936927	39948439	Zinc finger CCCH domain-containing protein 37-like
***qHSW_B05.1***	Araip.B05	*Araip.W1XHN*	26367457	26370442	Protein YLS7-like
	Araip.B05	*Araip.QEE5K*	26463556	26466288	Serine/threonine-protein phosphatase
	Araip.B05	*Araip.8GK8W*	26024832	26025395	Protein FAR1-RELATED SEQUENCE 6-like isoform
	Araip.B05	*Araip.IJC5B*	26188466	26198447	Nodulin MtN21 /EamA-like transporter family protein
	Araip.B05	*Araip.FGM9R*	26261340	26263632	Acyl-CoA N-acyltransferases (NAT) superfamily protein
***qHSW_B06.1***	Araip.B06	*Araip.JGK52*	110857554	110860089	Spermidine hydroxycinnamoyl transferase-like
	Araip.B06	*Araip.Z5DXR*	111296004	111297328	Zinc-binding alcohol dehydrogenase family protein
	Araip.B06	*Araip.6N0ZN*	111333418	111334852	MYB transcription factor
	Araip.B06	*Araip.MK1X6*	111913983	111914486	Serine/threonine-protein phosphatase
	Araip.B06	*Araip.R3W9S*	112048940	112052397	E3 ubiquitin-protein ligase n
	Araip.B06	*Araip.86RJH*	112430911	112431479	CLP-similar protein
***qHSW_B09.1***	Araip.B09	*Araip.M2BYP*	145798830	145800398	Lipid transfer protein
	Araip.B09	*Araip.4HV2H*	145518107	145520042	Sugar transporter
	Araip.B09	*Araip.T9QAQ*	145492177	145493838	E3 ubiquitin-protein ligase
	Araip.B09	*Araip.U0WFW*	145740761	145742266	Seed maturation protein
	Araip.B09	*Araip.A4X5Z*	145725368	145727373	Cytochrome P450 superfamily protein
	Araip.B09	*Araip.9SE5V*	145953611	145955081	EPIDERMAL PATTERNING FACTOR-like protein 4-like
	Araip.B09	*Araip.D6PZJ*	145587000	145594367	Seed linoleate 9S-lipoxygenase
	Araip.B09	*Araip.Q00X2*	145593792	145596313	Spermidine synthase
	Araip.B09	*Araip.YK09Y*	145971775	145975639	Protein TIFY 4B-like isoform (*BIG SEED locus*)
***QShP_B10.1***	Araip.B10	*Araip.B3EI3*	133700271	133702677	Calcium-dependent lipid-binding (CaLB domain) family protein
	Araip.B10	*Araip.U1RD3*	133778488	133782909	Cellulose synthase
	Araip.B10	*Araip.RHZ53*	135758161	135760450	Cold acclimation protein *WCOR413* family
	Araip.B10	*Araip.P42EJ*	134195386	134198229	Disease resistance protein
	Araip.B10	*Araip.XJC3R*	133165446	133172336	E3 ubiquitin-protein ligase *RGLG2*-like isoform X4
	Araip.B10	*Araip.EJ1RQ*	135156885	135158036	F-box family protein
	Araip.B10	*Araip.I3ZKC*	135136304	135136707	LRR and NB-ARC domain disease resistance protein
	Araip.B10	*Araip.UA0W9*	133594505	133595933	NAC domain protein
	Araip.B10	*Araip.JW7JM*	135500237	135501639	Receptor-like protein kinase-like
	Araip.B10	*Araip.I4J8B*	134451076	134452602	Serine/threonine-protein phosphatase long form homolog
	Araip.B10	*Araip.3H2LW*	134153729	134154083	Sugar transporter SWEET

### 3.9 Expression of potential candidate genes at seed and pod developmental stages

**FIGURE 6:**
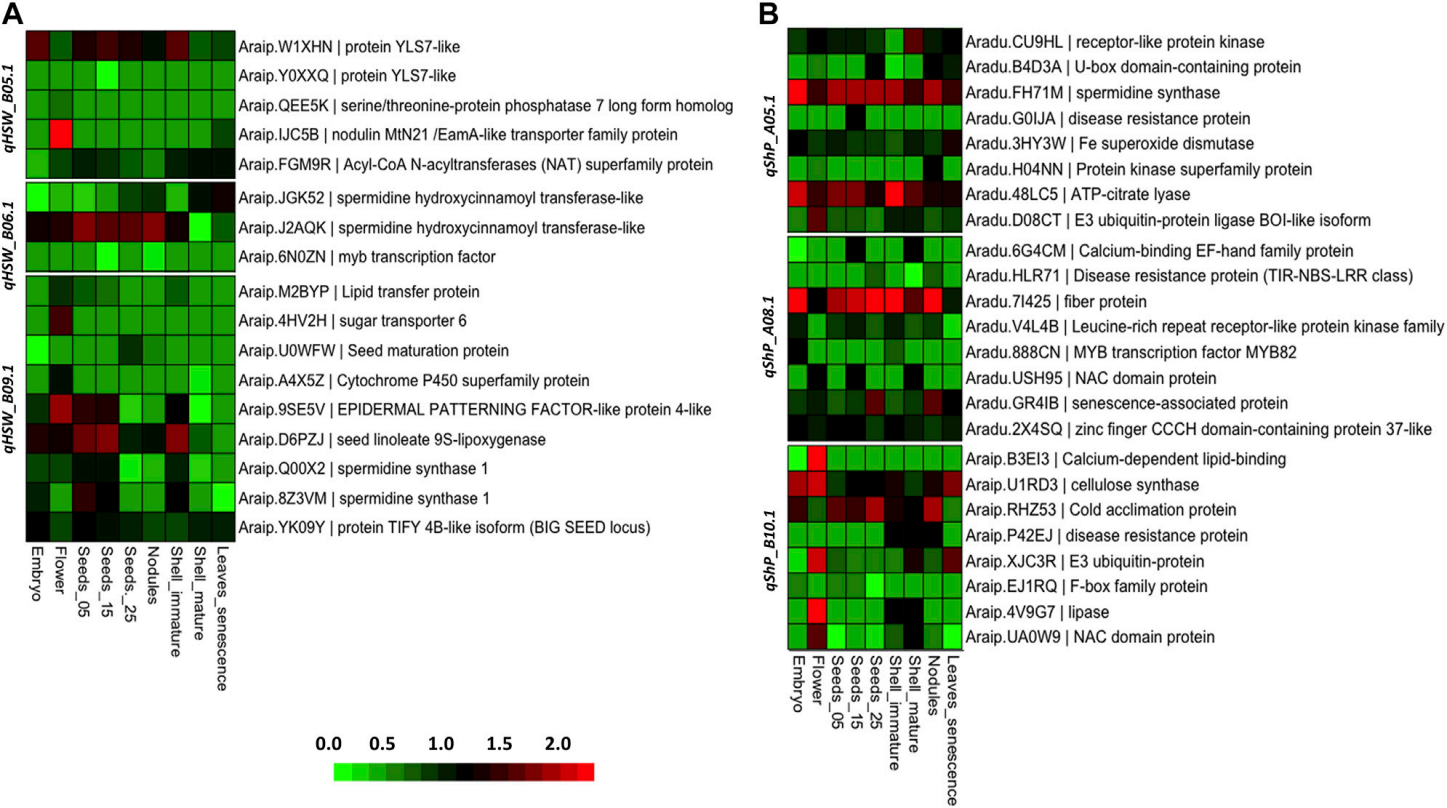
Heatmap illustrating the expression of candidate genes in various seed and pod development stages. **(A)** Heatmap for differential gene expression of genes identified in HSW QTL regions. **(B)** Heatmap for differential gene expression of genes identified in SHP QTL regions.

The two parents used for developing the RIL population were subsp. *fastigiata*; hence, we used the *fastigiata* gene expression atlas (Sinha et al., 2020) to study the tissue-specific expression of candidate genes identified for HSW and SHP. In the QTL region *qHSW_B05.1*, YSL-like protein (*Araip.W1XHN*) was highly expressed in embryo and seed tissues. We identified two isoforms of YSL-like protein (*Araip.W1XHN* and *Araip.Y0XXQ*). Acyl-CoA acyltransferase (*Araip.FGM9R*) showed high expression in pod walls as compared to seed developmental stages. From the QTL region (*qHSW_B06.1*), two isoforms of *spermidine synthase* (*Araip.JGK52* and *Araip.J2AQK*) showed contrasting expression. The expression of *Araip.J2AQK* was higher in flower embryo and seed developmental stages. In the QTL region (*qHSW_B09.1*), sugar transporters (*Araip.4HV2H*) were highly expressed in flowers. Seed maturation protein (*Araip.U0WFW*) was expressed at the time of maturity in seeds. The TIFY family protein (*BIG SEED* locus) (*Araip.YK09Y*) was highly expressed in embryo and seed developmental stages. The epidermal patterning factor (*Araip.9SE5V*) showed high expression in flowers, seeds, and shells. Seed linoleate 9S-lipoxygenase (*Araip.9SE5V*) is shown in Figure 6A (Supplementary Table S5).

In the case of shelling percentage, several disease-resistance genes were highly expressed, such as MYB, NBS-LRR, and NAC domain proteins. In the QTL region *qShP_A05.1*, the *spermidine synthase* (*Araip.8Z3VM*) and ATP citrate lyase (*Aradu.48LC5*) showed higher expression in all the seed and pod tissues. In the QTL region *qShP_A08.1*, the genes involved in the synthesis of cellulose were highly expressed in the SHP QTLs. For instance, cellulose synthase (*Aradu.GKF95*) and fibre proteins (*Aradu.7I425*) showed high expression in all seed and pod developmental stages. Senescence-associated protein (*Aradu.GR4IB*) was highly expressed in pod walls at the time of maturity. In the QTL region *qShP_B10.1*, cold acclimation protein (*Araip.RHZ53*) was highly expressed in seed and pods. The lipases (*Araip.4V9G7*) and NAC domains (*Araip.UA0W9*) were highly expressed in flowers and mature shells. The expression analysis of genes showed that there are key genes involved in the cellulose biosynthesis pathway. However, a group of disease-resistant genes were highly expressed in all SHP QTL regions (Figure 6B; Supplementary Table S6).

### 3.10 Development of KASP markers for hundred-seed weight in groundnut

We used multiple approaches for genetic dissection of groundnut seed weight. In a previous study, we used NAM population to map the genomic regions associated with seed weight and pod weight in groundnut (Gangurde et al., 2020). A KASP marker (snpAH00173) on chromosome A05 at 101618480 bp was developed and validated on small- and large-seeded groundnut germplasms. A sequencing-based trait mapping approach “QTL-seq” was also used to identify the genomic regions for HSW of groundnut. An overlapping genomic region was identified on chromosome B09 in the present genetic mapping study and QTL-seq analysis for groundnut seed weight. A total of four KASP markers (snpAH0031, snpAH0033, snpAH0037, and snpAH0038) were recently developed from the same population using the QTL-seq approach (Gangurde et al., 2021). Because of high polymorphism, the seed weight KASP’s markers were included in the quality control panel for their use in confirmation of F_1_s and hybrid purity testing. Moreover, the KASP markers can also be used in the marker-assisted selection breeding programs to improve the seed-weight trait in important groundnut cultivars. In this study, we discovered a novel genomic region on chromosome B09, containing important genes, such as *BIG SEED* locus and *spermidine synthase* (*spds*), associated with seed development.

## 4 Discussion

In the present study, a RIL population (Chico × ICGV 02251) was used for mapping the genomic regions associated with HSW and SHP in groundnut. The three seasons of phenotyping data and SNP array-based genotyping data were generated to identify the QTLs linked with HSW and SHP. We observed that HSW was comparatively higher in post-rainy seasons than in rainy seasons as confirmed by repeated planting in two consecutive rainy seasons. This might be due to high disease pressure in rainy seasons that affects seed size in groundnut. A high-density 58K SNP array was used to construct a dense genetic map comprising 4199 SNP loci in a map distance of 2708.36 cM with an average inter-marker distance of 0.65 cM. Only 7.2% of SNP loci (4199 SNPs out of 58,233 SNPs on the array) were mapped on 20 linkage groups of groundnut. Genetic diversity analysis in groundnut reported that it has a very narrow genetic base; therefore, the construction of very high-density genetic map in groundnut is very challenging (Pandey et al., 2012). The density of this genetic map was the highest when compared to the previous genetic maps constructed using the SNP array (Pandey M. K. et al., 2020) and genotyping by sequencing (GBS) (Dodia et al., 2019; Jadhav et al., 2021). Moreover, a genetic map with 3630 markers grouped in 2636 bins was used to identify the QTLs for groundnut seed weight (Wang et al., 2018). Genome-wide association analysis with DArT markers identified nine marker–trait associations for seed length and five for HSW, but due to the unavailability of annotated reference genome for groundnut, the researchers could not reach to candidate genes associated with HSW (Pandey et al., 2014). Earlier genotyping by SSR markers was laborious and time consuming. Now, allelic SNP markers along with high-quality reference genomes allows for genetic dissection of complex traits and the identification of candidate genes (Bertioli et al., 2019; Chen et al., 2019; Zhuang et al., 2019). In our previous study, we successfully used the SNP array for mapping the genomic regions associated with seed and pod weight in groundnut using NAM population (Gangurde et al., 2020). The seed size QTLs on chromosomes A05 and A07 were reported from two RIL populations (Luo et al., 2017; Luo et al., 2018). Interestingly, in this study, we identified a consistent major-effect QTL for shelling percentage on chromosome A05 with >10% PVE, and a major QTL on chromosome B05 was identified with 21% PVE with 7.7 LOD. In addition, a major-effect QTL was identified on chromosome B09 with 13.0% and 11% PVE. Therefore, the genomic region on chromosome B09 was targeted for the identification of candidate genes using PeanutBase (www.peanutbase.org).

We also demonstrated that the major effect of a QTL is not just because of the genetic background; sometimes, it might be due to the environmental effect or a combination of these two. A major QTL for HSW on chromosome B05 and two major QTLs for shelling percentage on chromosome B06 and B10 exhibited major additive by environmental effect (Q × E). Similar findings have been reported on the background effect and QTL × Environment effect for yield traits in rice (Wang et al., 2014).

Genes, such as *BIG SEED* locus and *spermidine synthase*, located in the QTL region on chromosome B09 negatively regulates the seed weight. Gene cloning for *BIG SEED* locus has been reported in *Medicago truncatula*, and the *BIG SEED* gene was isolated from soybean and overexpressed in the model legume crop, *Medicago truncatula*, which resulted in small seeds in transgenic lines (Ge et al., 2016). *Spermidine synthase* is also reported as the negative regulator of seed weight and size in rice (Tao et al., 2018). Therefore, we concluded that the *BIG SEED* locus and *spermidine synthase* genes can be targeted for the genome editing to enhance the groundnut seed weight. In the QTL region on chromosome B09, genes such as lipid transfer protein, sugar transporter, seed maturation protein, epidermal patterning factor-like proteins, and seed linoleate 9S-lipoxygenase associated with seed growth and development were identified. Seed linoleate 9S-lipoxygenase is a fat-metabolising gene linked with seed oil content (Wang et al., 2016). The epidermal patterning factor-like proteins are associated with plant epidermal cell growth factors and widely reported as a regulator for plant growth and development (Endo and Torii, 2019). A gene, serine threonine protein phosphatase identified in almost all QTL regions of seed weight and shelling percentage is associated with reactive oxygen species metabolism (ROS), plant’s cold tolerance, and abscisic acid signalling (Hou et al., 2016). We identified a copy of *spermidine synthase* in the QTL region (*QShP_A05*), identified for the shelling percentage on chromosome A05, along with receptor-like kinases (RLKs) which play a major role in plant growth and stress response (Cui et al., 2021).

In the present study, we identified several disease-resistance genes, such as disease-resistance protein (*Aradu.G0IJA*) and Leucine-rich repeat receptor-like protein (*Aradu.V4L4B*), that are from NBS-LRR class in the QTL regions of shelling percentage. In addition, MLO-like proteins, laccase, senescence-associated proteins, cold acclimation proteins, and F-box family proteins were identified in the QTL regions of shelling percentage. The groundnut shell is made up of cellulose and fibre; cellulose synthase, fibre proteins, and laccase were identified in the QTL regions for shelling percentage. SWEET genes encoding sugar transporters play a major role for plant growth and development (Gupta, 2020).

In this study, both parental genotypes used in developing the RIL population were from subsp. *fastigiata*. Therefore, we used a gene expression atlas developed from subsp. *fastigiata* (Sinha et al., 2020). From gene expression patterns, we observed that the isoforms of YSL7-like protein showed multiple gene-expression patterns. For instance, isoform *Araip.W1XHN* was highly expressed, and *Araip.Y0XXQ* was not expressed in seed and pod tissues. We identified multiple isoforms of *spermidine synthases* (*Araip.Q00X2* and *Araip.8Z3VM*) in the major QTLs of seed weight. The *BIG SEED* locus encoded by protein TIFY 4B-like isoform showed high expression in all seed developmental stages. In addition, we observed that the seed linoleate 9s-lipoxygenase (*Araip.D6PZJ*) and epidermal patterning factor-like proteins (*Araip.9SE5V*) are the most expressed genes in the seed tissues (Figure 6A). In the case of shelling percentage, the disease-resistance genes in QTL regions of shelling percentage were confirmed with gene expression atlas. Almost all disease-resistance genes in the QTL regions of shelling percentage were highly expressed in pod walls and seed tissues. Interestingly, the *spermidine synthase* genes were identified in the QTL regions of both seed weight and shelling percentage. At the time of maturity, the senescence-associated protein (*Araip.GR4IB*) was highly expressed in pod walls. Cellulose synthase and calcium-binding proteins NAC domains were differentially expressed in seed and pod tissues.

The identified genes, particularly, *spermidine synthase*, *BIG SEED* locus, and seed linoleate 9s lipoxygenase genes, can be targeted for functional validation and can be used in improving the seed weight and shelling percentage in groundnut. Furthermore, the QTLs will be validated on diverse seed weight groundnut genotypes for their use in genomics-assisted breeding for improving HSW and SHP.

## Data Availability

The original contributions presented in the study are included in the article/Supplementary Material, further inquiries can be directed to the corresponding author.

## References

[B1] Agarwal G. Clevenger J. Kale S. M. Wang H. Pandey M. K. Choudhary D. (2019). A recombination bin-map identified a major QTL for resistance to Tomato Spotted Wilt Virus in peanut (*Arachis hypogaea*). Sci. Rep. 9 (1), 18246–18313. 10.1038/s41598-019-54747-1 31796847 PMC6890646

[B2] Agarwal G. Clevenger J. Pandey M. K. Wang H. Shasidhar Y. Chu Y. (2018). High-density genetic map using whole-genome resequencing for fine mapping and candidate gene discovery for disease resistance in peanut. Plant Biotechnol. J. 16 (11), 1954–1967. 10.1111/pbi.12930 29637729 PMC6181220

[B3] Bailey W. K. Hammons R. O. (1975). Registration of Chico peanut germplasm 1 (reg. No. GP 2). Crop Sci. 15 (1), 105. 10.2135/cropsci1975.0011183x001500010050x

[B4] Ballén-Taborda C. Chu Y. Ozias-Akins P. Timper P. Holbrook C. C. Jackson S. A. (2019). A new source of root-knot nematode resistance from Arachis stenosperma incorporated into allotetraploid peanut (*Arachis hypogaea*). Sci. Rep. 9, 17702. 10.1038/s41598-019-54183-1 31776412 PMC6881346

[B5] Bertioli D. J. Cannon S. B. Froenicke L. Huang G. Farmer A. D. Cannon E. K. S. (2016). The genome sequences of *Arachis duranensis* and *Arachis ipaensis*, the diploid ancestors of cultivated peanut. Nat. Genet. 48 (4), 438–446. 10.1038/ng.3517 26901068

[B6] Bertioli D. J. Jenkins J. Clevenger J. Dudchenko O. Gao D. Seijo G. (2019). The genome sequence of segmental allotetraploid peanut *Arachis hypogaea* . *Arachis hypogaea*. Nat. Genet. 51 (5), 877–884. 10.1038/s41588-019-0405-z 31043755

[B7] Chavarro C. Chu Y. Holbrook C. Isleib T. Bertioli D. Hovav R. (2020). Pod and seed trait QTL identification to assist breeding for peanut market preferences. G3 Genes, Genomes, Genet. 10 (7), 2297–2315. 10.1534/g3.120.401147 PMC7341151 32398236

[B8] Chen W. Jiao Y. Cheng L. Huang L. Liao B. Tang M. (2016). Quantitative trait locus analysis for pod-and kernel-related traits in the cultivated peanut (*Arachis hypogaea* L.) BMC Genet. 17 (1), 25–29. 10.1186/s12863-016-0337-x 26810040 PMC4727316

[B9] Chen X. Lu Q. Liu H. Zhang J. Hong Y. Lan H. (2019). Sequencing of cultivated peanut, *Arachis hypogaea*, yields insights into genome evolution and oil improvement. Mol. Plant 12 (7), 920–934. 10.1016/j.molp.2019.03.005 30902685

[B10] Chen Y. Ren X. Zheng Y. Zhou X. Huang L. Yan L. (2017). Genetic mapping of yield traits using RIL population derived from Fuchuan Dahuasheng and ICG6375 of peanut (*Arachis hypogaea* L.) Mol. Breed. 37 (2), 17–14. 10.1007/s11032-016-0587-3 28216998 PMC5285419

[B11] Chu Y. Chee P. Culbreath A. Isleib T. G. Holbrook C. C. Ozias-Akins P. (2019). Major QTLs for resistance to early and late leaf spot diseases are identified on chromosomes 3 and 5 in peanut (*Arachis hypogaea* L.) Front. Plant Sci. 10 10, 883. 10.3389/fpls.2019.00883 p.883 PMC6625158 31333711

[B12] Chu Y. Chee P. Isleib T. G. Holbrook C. C. Ozias-Akins P. (2020). Major seed size QTL on chromosome A05 of peanut *(Arachis hypogaea)* is conserved in the US mini core germplasm collection. Mol. Breed. 40 (1), 6–16. 10.1007/s11032-019-1082-4

[B13] Clevenger J. Chu Y. Scheffler B. Ozias-Akins P. (2016). A developmental transcriptome map for allotetraploid *Arachis hypogaea* . *Arachis hypogaea*. Front. Plant Sci. 7, 1446. 10.3389/fpls.2016.01446 27746793 PMC5043296

[B14] Corley R. H. (2009). How much palm oil do we need? Environ. Sci. Policy 12 (2), 134–139. 10.1016/j.envsci.2008.10.011

[B15] Cui Y. Lu X. Gou X. (2021). Receptor-like protein kinases in plant reproduction: Current understanding and future perspectives. Plant Commun. 100273 3, 100273. 10.1016/j.xplc.2021.100273 PMC8760141 35059634

[B16] Dodia S. M. Joshi B. Gangurde S. S. Thirumalaisamy P. P. Mishra G. P. Narandrakumar D. (2019). Genotyping-by-sequencing based genetic mapping reveals large number of epistatic interactions for stem rot resistance in groundnut. Theor. Appl. Genet. 132 (4), 1001–1016. 10.1007/s00122-018-3255-7 30539317

[B17] Endo H. Torii K. U. (2019). Stomatal development and perspectives toward agricultural improvement. Cold Spring Harb. Perspect. Biol. 11 (5), a034660. 10.1101/cshperspect.a034660 30988007 PMC6496345

[B18] Fonceka D. Tossim H. A. Rivallan R. Vignes H. Faye I. Ndoye O. (2012). Fostered and left behind alleles in peanut: Interspecific QTL mapping reveals footprints of domestication and useful natural variation for breeding. BMC Plant Biol. 12 (1), 26–16. 10.1186/1471-2229-12-26 22340522 PMC3312858

[B19] Food and Agriculture Organization of the United Nations Database (FAOSTAT) (2018). Online database at: https://www.fao.org/faostat/en/#data March Accessed on 23, 2022).

[B20] Gangurde S. S. Kumar R. Pandey A. K. Burow M. Laza H. E. Nayak S. N. (2019). Climate-smart groundnuts for achieving high productivity and improved quality: Current status, challenges, and opportunities. Genomic Des. climate-smart oilseed crops, 133–172. 10.1007/978-3-319-93536-2_3

[B21] Gangurde S. S. Nayak S. N. Joshi P. Purohit S. Sudini H. K. Chitikineni A. (2021). Comparative transcriptome analysis identified candidate genes for late leaf spot resistance and cause of defoliation in groundnut.*Int* . *J. Mol. Sci.*22(9) 4491. 10.3390/ijms22094491 PMC8123497 33925801

[B22] Gangurde S. S. Wang H. Yaduru S. Pandey M. K. Fountain J. C. Chu Y. (2020). Nested-association mapping (NAM)-based genetic dissection uncovers candidate genes for seed and pod weights in peanut (*Arachis hypogaea*). Plant Biotechnol. J. 18 (6), 1457–1471. 10.1111/pbi.13311 31808273 PMC7206994

[B23] Ge L. Yu J. Wang H. Luth D. Bai G. Wang K. (2016). Increasing seed size and quality by manipulating *BIG SEEDS1* in legume species. Proc. Natl. Acad. Sci. 113 (44), 12414–12419. 10.1073/pnas.1611763113 27791139 PMC5098654

[B24] Gupta P. K. (2020). SWEET genes for disease resistance in plants. Trends Genet. 36 (12), 901–904. 10.1016/j.tig.2020.08.007 32896434

[B25] Han S. Yuan M. Clevenger J. P. Li C. Hagan A. Zhang X. (2018). A SNP-based linkage map revealed QTLs for resistance to early and late leaf spot diseases in peanut (*Arachis hypogaea* L.) Front. Plant Sci. 9, 10.3389/fpls.2018.01012. 10.3389/fpls.2018.01012 PMC6048419 30042783

[B26] Hou Y. J. Zhu Y. Wang P. Zhao Y. Xie S. Batelli G. (2016). Type one protein phosphatase 1 and its regulatory protein inhibitor 2 negatively regulate ABA signaling. PLoS Genet. 12 (3), e1005835. 10.1371/journal.pgen.1005835 26943172 PMC4778861

[B27] Jadhav M. P. Gangurde S. S. Hake A. A. Yadawad A. Mahadevaiah S. S. Pattanashetti S. K. (2021). Genotyping-by-sequencing based genetic mapping identified major and consistent genomic regions for productivity and quality traits in peanut. Front. Plant Sci. 12, 668020. 10.3389/fpls.2021.668020 2034 34630444 PMC8495222

[B28] Kolde R. (2019). Pheatmap: Pretty heatmaps version 1.0.12. Available at: https://cran.r-project.org/web/packages/pheatmap/index.html .

[B29] Kosambi D. D. (1944). The estimation of map distances from recombination values. Ann. Eugen. 12, 172–175. 10.1111/j.1469-1809.1943.tb02321.x

[B30] Kumar R. Janila P. Vishwakarma M. K. Khan A. W. Manohar S. S. Gangurde S. S. (2020). Whole-genome resequencing-based QTL-seq identified candidate genes and molecular markers for fresh seed dormancy in groundnut. Plant Biotechnol. J. 18 (4), 992–1003. 10.1111/pbi.13266 31553830 PMC7061874

[B31] Luo H. Guo J. Ren X. Chen W. Huang L. Zhou X. (2018). Chromosomes A07 and A05 associated with stable and major QTLs for pod weight and size in cultivated peanut (*Arachis hypogaea* L.) Theor. Appl. Genet. 131 (2), 267–282. 10.1007/s00122-017-3000-7 29058050

[B32] Luo H. Pandey M. K. Khan A. W. Guo J. Wu B. Cai Y. (2019a). Discovery of genomic regions and candidate genes controlling shelling percentage using QTL-seq approach in cultivated peanut (*Arachis hypogaea* L.) Plant Biotechnol. J. 17 (7), 1248–1260. 10.1111/pbi.13050 30549165 PMC6576108

[B33] Luo H. Pandey M. K. Khan A. W. Wu B. Guo J. Ren X. (2019b). Next-generation sequencing identified genomic region and diagnostic markers for resistance to bacterial wilt on chromosome B02 in peanut (*Arachis hypogaea* L.) Plant Biotechnol. J. 17 (12), 2356–2369. 10.1111/pbi.13153 31087470 PMC6835129

[B34] Luo H. Ren X. Li Z. Xu Z. Li X. Huang L. (2017). Co-localization of major quantitative trait loci for pod size and weight to a 3.7 cM interval on chromosome A05 in cultivated peanut (*Arachis hypogaea* L.) BMC Genom. 18 (1), 58–12. 10.1186/s12864-016-3456-x PMC5223410 28068921

[B35] Luo Z. Cui R. Chavarro C. Tseng Y. C. Zhou H. Peng Z. (2020). Mapping quantitative trait loci (QTLs) and estimating the epistasis controlling stem rot resistance in cultivated peanut (*Arachis hypogaea*). Theor. Appl. Genet. 133 (4), 1201–1212. 10.1007/s00122-020-03542-y 31974667

[B36] Meng L. Li H. Zhang L. Wang J. (2015). QTL IciMapping: Integrated software for genetic linkage map construction and quantitative trait locus mapping in biparental populations. Crop J. 3, 269–283. 10.1016/j.cj.2015.01.001

[B37] Mondal S. Badigannavar A. M. (2019). Identification of major consensus QTLs for seed size and minor QTLs for pod traits in cultivated groundnut (*Arachis hypogaea* L.) 3 Biotech. 9 (9), 347–349. 10.1007/s13205-019-1881-7 PMC6717230 31497465

[B38] Nabi R. B. S. Cho K. S. Tayade R. Oh K. W. Lee M. H. Kim J. I. (2021). Genetic diversity analysis of Korean peanut germplasm using 48 K SNPs ‘Axiom_Arachis’ Array and its application for cultivar differentiation. Sci. Rep. 11 (1), 16630. 10.1038/s41598-021-96074-4 34404839 PMC8371136

[B61] Pandey M. K. Khan A. W. Singh V. K. Vishwakarma M. K. Shasidhar Y. Kumar V. (2017a). QTL-seq approach identified genomic regions and diagnostic markers for rust and late leaf spot resistance in groundnut (*Arachis hypogaea* L.) Plant. Biotechnol. J. 15 (8), 927–941. 28028892 10.1111/pbi.12686 PMC5506652

[B39] Pandey M. K. Agarwal G. Kale S. M. Clevenger J. Nayak S. N. Sriswathi M. (2017b). Development and evaluation of a high-density genotyping ‘Axiom_Arachis’ array with 58 K SNPs for accelerating genetics and breeding in groundnut. Sci. Rep. 7 (1), 40577–40610. 10.1038/srep40577 28091575 PMC5238394

[B40] Pandey M. K. Pandey A. K. Kumar R. Nwosu C. V. Guo B. Wright G. C. (2020a). Translational genomics for achieving higher genetic gains in groundnut. Theor. Appl. Genet. 133 (5), 1679–1702. 10.1007/s00122-020-03592-2 32328677 PMC7214508

[B42] Pandey M. K. Gangurde S. S. Sharma V. Pattanashetti S. K. Naidu G. K. Faye I. (2020b). Improved genetic map identified major QTLs for drought tolerance-and iron deficiency tolerance-related traits in groundnut. Genes 12 (1), 37. 10.3390/genes12010037 33396649 PMC7824586

[B41] Pandey M. K. Monyo E. Ozias-Akins P. Liang X. Guimarães P. Nigam S. N. (2012). Advances in Arachis genomics for peanut improvement. Biotechnol. Adv. 30 (3), 639–651. 10.1016/j.biotechadv.2011.11.001 22094114

[B43] Pandey M. K. Upadhyaya H. D. Rathore A. Vadez V. Sheshshayee M. S. Sriswathi M. (2014). Genome-wide association studies for 50 agronomic traits in peanut using the ‘reference set’ comprising 300 genotypes from 48 countries of the semi-arid tropics of the world. PLoS one 9 (8), e105228. 10.1371/journal.pone.0105228 25140620 PMC4139351

[B45] Parmar S. Sharma V. Bomireddy D. Soni P. Joshi P. Gangurde S. S. (2022). Recent advances in genetics, genomics, and breeding for nutritional quality in groundnut. Accel. Plant Breed. 4, 111–137.

[B46] Shasidhar Y. Variath M. T. Vishwakarma M. K. Manohar S. S. Gangurde S. S. Sriswathi M. (2020). Improvement of three popular Indian groundnut varieties for foliar disease resistance and high oleic acid using SSR markers and SNP array in marker-assisted backcrossing. Crop J. 8 (1), 1–15. 10.1016/j.cj.2019.07.001

[B47] Sinha P. Bajaj P. Pazhamala L. T. Nayak S. N. Pandey M. K. Chitikineni A. (2020). *Arachis hypogaea* gene expression atlas for *fastigiata* subspecies of cultivated groundnut to accelerate functional and translational genomics applications. Plant Biotechnol. J. 18 (11), 2187–2200. 10.1111/pbi.13374 32167667 PMC7589347

[B48] Tao Y. Wang J. Miao J. Chen J. Wu S. Zhu J. (2018). The *spermine synthase OsSPMS1* regulates seed germination, grain size, and yield. Plant Physiol. 178 (4), 1522–1536. 10.1104/pp.18.00877 30190417 PMC6288755

[B49] Varshney R. K. Bertioli D. J. Moretzsohn M. de C. Vadez V. Krishnamurthy L. (2009). The first SSR-based genetic linkage map for cultivated groundnut (*Arachis hypogaea* L.) Theor. Appl. Genet. 118 (4), 729–739. 10.1007/s00122-008-0933-x 19048225

[B50] Voorrips R. (2002). MapChart: Software for the graphical presentation of linkage maps and QTLs. J. Hered. 93, 77–78. 10.1093/jhered/93.1.77 12011185

[B51] Wang M. L. Wang H. Zhao C. Tonnis B. Tallury S. Wang X. (2022). Identification of QTLs for seed dormancy in cultivated peanut using a recombinant inbred line mapping population. Plant Mol. Biol. Rep. 40, 208–217. 10.1007/s11105-021-01315-5

[B52] Wang X. Pang Y. Zhang J. Zhang Q. Tao Y. Feng B. (2014). Genetic background effects on QTL and QTL× environment interaction for yield and its component traits as revealed by reciprocal introgression lines in rice. Crop J. 2 (6), 345–357. 10.1016/j.cj.2014.06.004

[B53] Wang Y. Ma X. Zhang X. He X. Li H. Cui D. (2016). ITRAQ-based proteomic analysis of the metabolic mechanisms behind lipid accumulation and degradation during peanut seed development and post-germination. J. Proteome Res. 15 (12), 4277–4289. 10.1021/acs.jproteome.6b00345 27669742

[B54] Wang Z. Huai D. Zhang Z. Cheng K. Kang Y. Wan L. (2018). Development of a high-density genetic map based on specific length amplified fragment sequencing and its application in quantitative trait loci analysis for yield-related traits in cultivated peanut. Front. Plant Sci. 9, 827. 10.3389/fpls.2018.00827 29997635 PMC6028809

[B55] Zhang H. Chu Y. Dang P. Tang Y. Jiang T. Clevenger J. P. (2020). Identification of QTLs for resistance to leaf spots in cultivated peanut (*Arachis hypogaea* L.) through GWAS analysis. Theor. Appl. Genet. 133 (7), 2051–2061. 10.1007/s00122-020-03576-2 32144466

[B56] Zhang S. Hu X. Miao H. Chu Y. Cui F. Yang W. (2019). QTL identification for seed weight and size based on a high-density SLAF-seq genetic map in peanut (*Arachis hypogaea* L.) BMC Plant Biol. 19 (1), 1–15. 10.1186/s12870-019-2164-5 31795931 PMC6892246

[B57] Zhuang W. Chen H. Yang M. Wang J. Pandey M. K. Zhang C. (2019). The genome of cultivated peanut provides insight into legume karyotypes, polyploid evolution and crop domestication. Nat. Genet. 51 (5), 865–876. 10.1038/s41588-019-0402-2 31043757 PMC7188672

[B58] Zou K. Kang D. Kim K. S. Jun T. H. (2020b). Screening of salinity tolerance and genome-wide association study in 249 peanut accessions (*Arachis hypogaea* L.) Plant Breed. Biotech. 8 (4), 434–438. 10.9787/pbb.2020.8.4.434

[B59] Zou K. Kim K. S. Kang D. Kim M. C. Ha J. Moon J. K. (2022). Genome-wide association study of leaf chlorophyll content using high-density SNP array in peanuts (*Arachis hypogaea* L.) Agronomy 12 (1), 152. 10.3390/agronomy12010152

[B60] Zou K. Kim K. S. Kim K. Kang D. Park Y. H. Sun H. (2020a). Genetic diversity and genome-wide association study of seed aspect ratio using a high-density SNP array in peanut (*Arachis hypogaea* L.) Genes 12 (1), 2. 10.3390/genes12010002 33375051 PMC7822046

