# Supplementary figures and images for: Genetic mapping identifies genomic regions and candidate genes for seed weight and shelling percentage in groundnut
Source: Front Genet. 2023 Mar 16;14:1128182. doi: 10.3389/fgene.2023.1128182 (PMC10061104; doi:10.3389/fgene.2023.1128182)

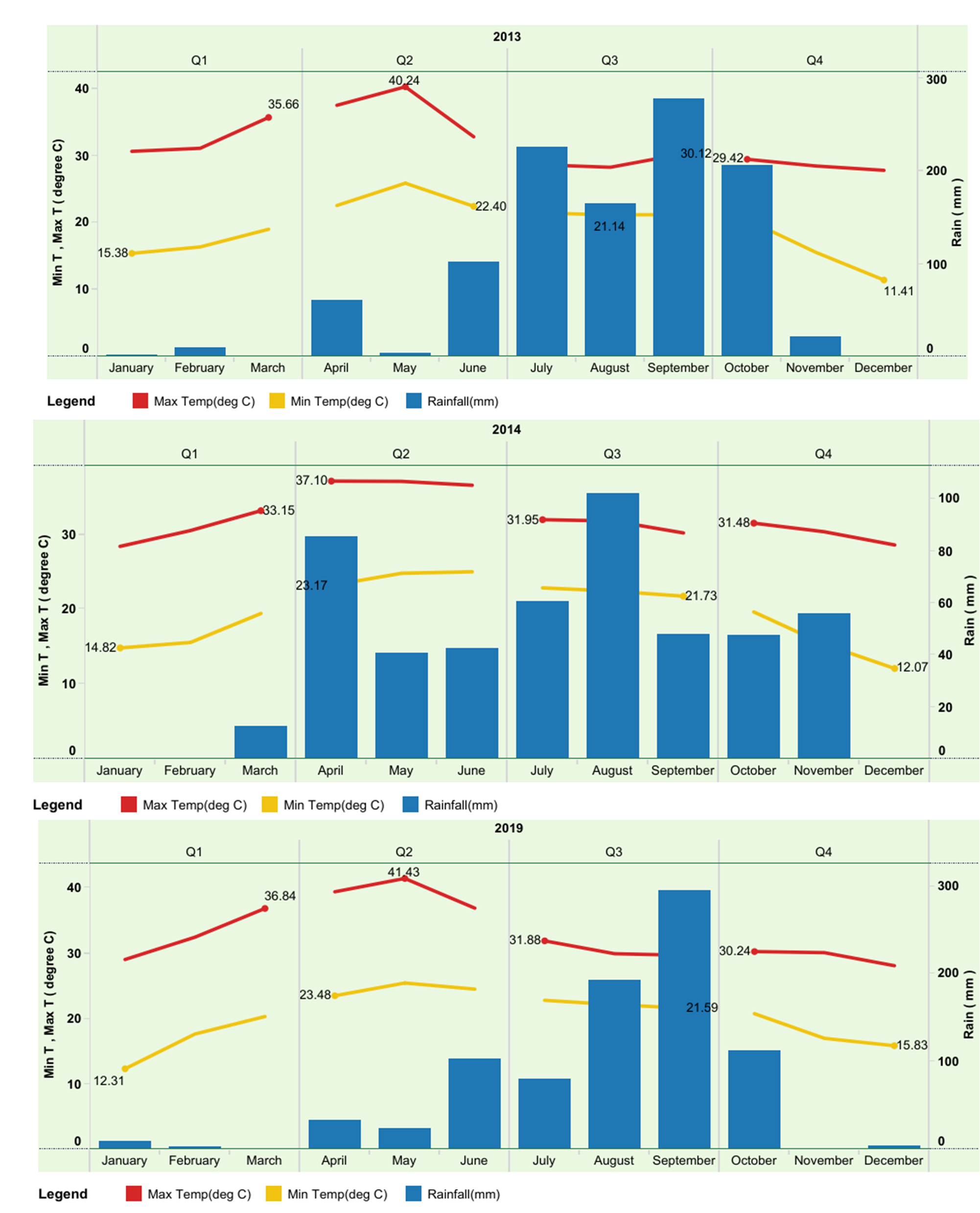

Supplement: Supplementary file 7 [file Image1.jpg]
